# Development and Implementation of an Ultrasound Wireless Technology Educational Program for Nursing Students: A Quality Improvement Project

**DOI:** 10.3390/nursrep15020063

**Published:** 2025-02-10

**Authors:** Elena Morotti, Sergio Rovesti, Chiara Diambri, Davide Boni, Rosaria Di Lorenzo, Dalia Caleffi, Mauro Bellifemine, Paola Ferri

**Affiliations:** Department of Biomedical, Metabolic and Neural Sciences, University of Modena and Reggio Emilia, 41125 Modena, Italy; elena.morotti@unimore.it (E.M.); sergio.rovesti@unimore.it (S.R.); diambrichiara0@gmail.com (C.D.); davide.boni@unimore.it (D.B.); saradilorenzo1@alice.it (R.D.L.); dalia.caleffi@unimore.it (D.C.); mauro.bellifemine@unimore.it (M.B.)

**Keywords:** nursing students, vascular access, ultrasound, ultrasound bladder scan, ultrasound inferior vena cava

## Abstract

**Background**: Training on the use of ultrasounds (US) is offered to nurses after their degree in specialization courses or in a work setting. When considering the positive impact of US on patient quality of care, this training should be offered to undergraduate nursing students. The aim of this quality improvement project was to assess the quality of nursing curricula by evaluating the effects of an ultrasound technology educational program (USTep) on nursing students’ knowledge, self-confidence, satisfaction and perceived usefulness of the training for the acquisition of US skills. **Methods**: 118 nursing students completed a 3-h USTep, that combined a theoretical introduction with simulation training. Data were collected before and after the USTep, using a survey with closed and open-ended questions. **Results**: After the educational program, a net increase was seen in knowledge about US (pre-test 48.1% vs. post-test 93.4%, *p* < 0.00001) and in student self-confidence (pre-test m = 1.7 ± 0.9 vs. post-test m = 3.9 ± 0.8, *p* < 0.001). According to the participants, this training benefited the students (during their training and for future employment opportunities), the patients, and the profession. Lastly, 97% of the sample expressed satisfaction with the training experience. **Conclusions**: This quality improvement project shows that a 3-h USTep improved undergraduate nursing students’ knowledge, self-confidence, and satisfaction.

## 1. Introduction

In recent years, ultrasounds (US) have become increasingly more useful in nursing practice since their use improves care outcomes as well as patient and nurse satisfaction [1,2,3]. Duplex ultrasound is a non-invasive procedure that allows healthcare-workers to see pictures of organs, tissues and blood flow in real time, using high-frequency ultra-sound waves and with color Doppler flow imaging [4]. The areas of application for nurses include the placement of vascular accesses, insertion of bladder catheters and nasogastric tubes, lung examination technique and monitoring urine volume, as well as examining the inferior vena cava (IVC).

Peripheral intravenous catheters (PIVs) are widely used medical devices in the healthcare field to join the surface of the skin to a peripheral venous vessel, for the administration of drugs, hydration, and parenteral nutrition, blood and derivatives [5]. A peripheral venous access device is inserted in approximately 85% of hospitalized patients and up to 90% of patients treated in Emergency Room [6,7]. In some situations, to place them correctly, several attempts at insertion are necessary. This condition, defined as difficult intravenous access (DIVA), occurs in 10–24% of hospitalized adults and entails an increased risk of adverse events (phlebitis, infiltration, extravasation, bruising, hemorrhages, infections, sepsis), discomfort, pain, anxiety, delays in treatment and prolonging the hospital stay [8,9,10]. The ensuing impoverishment of the vascular tree can lead to a “depletion of the peripheral venous heritage” and increase utilization of central venous access devices (CVADs), with increased morbidity, mortality and healthcare costs [11,12,13]. The European Society of Anaesthesiology (2020) recommends using ultrasounds for inserting PIVs with moderate or difficult access, both in emergency and elective cases [14]. Using ultrasounds by nurse permits assessing the position, diameter and depth of the vein to choose the best point of insertion and the appropriate length and diameter of the venous catheter, thereby promoting the preservation of peripheral veins, ensuring the integrity of vessel health. Some studies have shown how US improve the efficacy of the PIV insertion technique, in terms of number of attempts, time and success rate compared to the traditional method, reducing the need to resort to a CVAD from 74 to 85% [3,15,16,17,18,19]. Research evaluating the effectiveness of ultrasound guided PIV SBML (simulation-based mastery learning) observed that nurses trained using this method reported a success rate of 89.5% in cannulating venous accesses [20]. Ultrasound-guided PIV insertion is recommended in the following conditions: obesity, veins not visible or palpable, failure of 2 traditional attempts at IV catheter insertion, history of drug abuse, chronic conditions (e.g., diabetes or end-stage renal disease), chemotherapy, vascular disease and frequent hospitalizations [21]. In addition, nurses can choose to place a US-guided peripheral IV catheter according to the patient’s anxiety or because the patient requests it, because of limited access due to a greater number of peripheral catheters or a protracted hospital stay [21].

The use of ultrasound is also useful in facilitating arterial cannulation. Indeed, Li et al. (2016) state that, compared with the traditional method, radial artery cannulation with US is a quick and safe cannulation method suitable for use in the clinic [22]. Furthermore, a recent study demonstrates how emergency nurses can successfully use bedside ultrasound for radial artery puncture [23].

Rates of urinary catheter use are about 20% in non-intensive care areas and up to 60% in intensive care units, and catheter-associated urinary tract infections remain a relevant type of infection associated with healthcare [24]. Several studies highlighted that the non-invasiveness, cost-effectiveness, reliability and accuracy of using ultrasound as an alternative for invasive catheterization [24]. Bladder ultrasound helps the nurse to define and monitor the urinary volume of the bladder. It also reduces the time of urinary catheterization, avoids unnecessary catheterization and therefore prevents urinary tract infections [24,25,26].

Point-of-care ultrasound (POCUS) is a modern technique defined as bedside ultra-sound performed directly by a healthcare provider. In this methodology, in addition to the techniques already mentioned, nurses can perform the lung ultrasound scan, which enables early detection of signs of congestion in that area, allowing shorter waiting times for a medical examination and therefore better treatment [27,28]. Another field of US use is covered in the recent study of Kalam and colleagues, who confirmed that properly trained nurses can effectively use US to accurately assess the fluid status of septic patients, change the treatment plan originally envisioned by the physician, and increase physician confidence in the patient’s treatment plan [29]. A systematic review suggests that ultrasound of the inferior vena cava by registered nurses has the potential to assist in defining the ultrafiltration goal for that particular dialysis session, thus reducing the risk of intra-dialytic hypotension [30].

A nasogastric tube is frequently used for the purposes of nutrition, decompression or for administering medicines, whose dislocation can cause severe complications [31,32,33]. Ultrasound scanning is a fast, non-invasive and accurate method by which the nurse can assess gastric residual volume and nasogastric tube positioning, as an alternative method to radiography [34,35,36].

Ultrasound training is typically provided during post-basic training courses or in work settings. Considering the clinical importance and care benefits of using US it could be useful, right from basic training, to bring future nurses closer to this type of technology so as to promote its knowledge and use. To our knowledge, in literature there are no studies performed in the setting of undergraduate nursing programs.

The aim of this project was to design, implement and evaluate the effects of an ultra-sound technology educational program (USTep) on nursing students’ knowledge, self-confidence and satisfaction.

## 2. Materials and Methods

### 2.1. Design and Setting

This was an educational offer quality improvement (QI) project modeled prospective single arm pre- and post-study, following Rogers’ (2003) “Diffusion of Innovations” theoretical framework [37].

Rogers’ theory describes five characteristics of an innovation that make it acceptable for adoption and that project managers should consider when introducing innovative changes:

(1) Relative Advantage: the degree to which the innovation is perceived to be superior to current practice.
(2) Compatibility: the degree to which the innovation is perceived to be consistent with socio-cultural values, previous ideas, and/or perceived needs.
(3) Complexity: the degree to which an innovation is difficult to use or understand, its simplicity.
(4) Trialability: the degree to which the innovation can be experienced on a limited basis.
(5) Observability: the degree to which the results of an innovation are visible to potential adopters [38].

Considering the present study, these characteristics, assessed through informal interviews with stakeholders (students and expert trainers), were all largely satisfied.

Rogers also identifies five stages of the adoption process [38]:

(a) Knowledge or Awareness Stage: individual is exposed to innovation but lacks complete information. This stage is associated with the theoretical phase of the educational experience offered to students.
(b) Persuasion or Interest Stage: individual becomes interested in the new idea and seeks further information, just as the participants in the practical phase demonstrated.
(c) Decision or Evaluation Stage: individual mentally applies innovation to his present and anticipated future situation and then decides whether or not to try it. This mental approach to the innovation introduced can be associated with the reflections generated by the open-ended questions posed to students.
(d) Implementation or Trial Stage: individual makes full use of innovation.
(e) Confirmation or Adoption Stage: individual decides to continue the full use of innovation.

The two last phases are beyond the scope of this investigation but can be identified in the future studies proposed by the authors.

The QI project was conducted at the Nursing Program of the University of Modena and Reggio Emilia, located in Modena (Italy). The course is aimed at training a generalist nurse and is licensing to the profession. In Italy, standard nursing education programs last 3 years (180 ECTS) and the curricula is composed of theoretical teaching and practical training.

Before the implementation of this QI project, students did not receive any type of theoretical or practical training on the use of ultrasound.

### 2.2. Participants

124 undergraduate nursing students in their 3rd year of the nursing degree course during the Academic Year 2022/2023 were eligible for the study.

Inclusion criteria: the requirements for participation in the study were to be a student regularly enrolled in the third year of the course, to have provided adhesion to the study, and to have participated in the entire training experience entitled “Notes on nursing ultrasound: pelvic evaluation, evaluation of venous and arterial assets”.

Exclusion criteria: not being students enrolled in the third year of the course, not having provided adhesion to the study and not having participated in the training project during the period of investigation.

Since the QI project was totally innovative for the nursing degree course, and therefore it has never been tested within the nursing curriculum, it was initially proposed to the cohort of students who were better aware and prepared from a theoretical-practical point of view (a preparation provided within the third year of the course), so as to be able to evaluate the quality of the training offer, before its possible diffusion over all of the three years.

A power analysis was performed using G. Power 3.1.9.7 software to determine the sample size. By fixing the parameters as α = 0.05 and 1-β = 0.95, with an effect size = 0.5 on self-confidence, the minimum sample size was found to be 54 nursing students.

### 2.3. Ultrasound Wireless Technology Educational Program

The educational workshops on using an ultrasound scan in a nursing setting were conducted between February and April 2023 at the Advanced Training and Medical Simulation Center of the University. Instructors were nurses and mentors from the graduate program with post-basic training (master’s degree in critical care area) and specific courses in nursing ultrasound with subsequent bedside experience, as well as more than 10 years of clinical experience in the critical care area. The training consisted of a three-hour USTep divided into a theoretical introduction and a training simulation [39].

The educational activity began with a presentation of the physical basics of ultrasounds and their fields of use, the image interpretation, the various procedures that nurses can implement, and the equipment. Specifically, the instrument used was the GE HealthCare Vscan Air^TM^. On different days, 10 small groups composed of about 11 students carried out the practical exercise, in which each student was able to practice using the equipment to assess the peripheral venous patrimony, inferior vena cava, bladder volume and content and to the identification of arterial vessels, specifically one of the sites usually used in arterial blood gas sampling or arterial cannulation, i.e., the radial site, as well as the ulnar site for checking collateral circulation.

Before the practical exercise by the students, the instructors provided a demonstration of the correct use of the ultrasound scanner, showing the students how to perform the following practices: orientation of the probe, interpretation of the image in the procedures previously listed, use of the US Doppler.

Subsequently, students had the opportunity to experiment, under the supervision of the instructors, with the use of the technology on their classmates. The practical part lasted two hours, in which the instructors played the role of active observers to promote the learning of the ultrasound technique and encourage the correct use of the instrument.

### 2.4. Data Collection

The nursing educators developed this self-administered questionnaire after a literature review on US to assess nursing students’ knowledge, self-confidence and satisfaction. Subsequently, the tool was submitted to a restricted students and educators’ panel who were not involved in the QI project to assess the understandability of the tool.

The instrument was divided into four parts to investigate:

1. Sociodemographic characteristics of the students (gender, age and secondary education).
2. The theoretical knowledge learned by the students by means of a test of 5-item questions assessing knowledge of basic ultrasound use and indications, for which it was necessary to indicate the correct option from the ones presented.
3. Perceived self-confidence in relation to the use of the ultrasound scan by means of a test of 5-item statements with a 5-point Likert scale, ranging from 1 (Strongly disagree) to 5 (Strongly agree). The internal consistency measured with Cronbach’s alpha was 0.91 for this scale.
4. Qualitative data: two open-ended questions were also administered at the end of the educational activity to collect students’ feedback about training and assess their satisfaction: (a) Was educational intervention helpful in acquiring ultrasound technology competencies? and (b) What suggestions do you have to improve the educational intervention?
5. Students’ satisfaction was investigated by means of a closed-ended question with a 5-point Likert scale, ranging from 1 (Very dissatisfied) to 5 (Very satisfied).

The overall compilation time for all tools was approximately 20 min.

The data collection instrument was administered immediately before the start of the training and immediately at its completion, for Section 1, Section 2 and Section 3, while only at the end of the educational intervention for Section 4 and Section 5. Each student generated an identification code to be placed on the pre-test, so as to guarantee the confidentiality of the data provided, which will then be used in the post-test.

### 2.5. Statistical Analysis

The general characteristics of the sample were analyzed using descriptive: means (M) ± standard deviations (SD) and percentages. The Shapiro–Wilk test was employed to assess the normality of the study variables. Pre-test–post-test differences in study variables between the groups were analyzed with two-sample paired *t*-tests and Pearson’s chi2-test. In pre- and post-test, the homogeneity of the baseline characteristics (gender, age, and qualification) and the dependent variables (correctness of the answers provided and self-confidence) were assessed using the following tests: Pearson’s chi2-test, independent *t*-test, Pearson’s correlation, and ANOVA test. A *p*-value less than 0.05 was considered statistically significant. Statistical analyses were performed using SPSS Software Package version 29 (SPSS Inc., Chicago, IL, USA). A conventional content analysis was used to analyze the qualitative data and determine the presence of certain themes [40,41].

### 2.6. Ethical Considerations

This is an educational innovation project to improve the quality of training for future nurses, which was approved by the Department Council. It was conducted following the principles of the Declaration of Helsinki of the World Medical Association (2013) and the General Data Protection Regulation (Regulation EU 2016/679). All students received both oral and written information about the aim of this quality improvement project. In particular, students were informed about the voluntary nature of participation and that it would not affect their education or legal rights. They gave informed consent to participate when they answered the questionnaire. There was no form of coercion or undue influence on the students regarding participation. Participants were reassured about the confidentiality of the data collected. All responses were provided anonymously, as no personal data were requested or collected in the paper survey administered.

## 3. Results

### 3.1. Demographics

Out of a total of 124 eligible participants, 118 (95.2%) completed USTep. The mean age of the participants was 23.1 ± 3.7 SD years old, most of the participants were females (86%). Most of the sample (59%) had a high school diploma, 30% had a technical school diploma and the remainder had a vocational school diploma (11%). Only 2.5% of participants had a previously acquired bachelor’s degree.

### 3.2. Knowledge Results

As shown in Table 1, after training, a statistically significant increase was observed in theoretical knowledge for 4 out of 5 questions, while in one question the correct answer had already been indicated by a high percentage of respondents in the pre-test. Overall, the right answers after training passed from 48.1% to 93.4% (χ2 = 100.5, *p* < 0.00001).

Students identified the correct graphical representation of US in 98.3% of cases compared with 71.2% of correct answers in the pre-test. A significant increase was recorded for the question that sought to investigate whether learners were able to identify the correct order of magnitude of frequencies commonly used in clinical practice, rising from only 4 correct answers in the pre-test (3.4%) to 95 in the post-test (80.5%). The students, on the other hand, appeared to be very prepared prior to training on the aspects that make nursing ultrasound a reliable method; in fact, the change between before and after training was found to be only 1.65 percentage points, registering no statistical significance. In the post-test, the respondent cohort was found to be very prepared in correctly identifying the correct probe to be used for venous access retrieval; as can be seen, only 5 out of 118 students were unable to answer correctly after the training they received. A statistically significant increase was also recorded for the question that asked to identify the characteristics of pelvic nursing ultrasound, and 93.2% of the sample understood that it is not conducted for diagnostic purposes.

No differences emerged in the correctness of the answers given in relation to gender, age groups or qualification held (Pearson’s chi2 -test).

### 3.3. Self-Confidence Results

As shown in Table 2, participant confidence levels improved in a statistically significant way, after the educational program, in all five statements. The mean of the self-confidence scores in the pre-test grew from 1.7 ± 0.9 to 3.9 ± 0.8 in the post-test, with an increase of 2.2 points on a 5-point Likert scale (*t*-test = −27.32, *p* < 0.001). No differences emerged in mean total score of self-confidence, in pre and in post-test, in relation to gender (independent *t*-test), age (Pearson’s correlation) or qualification held (ANOVA test).

### 3.4. Qualitative Results

As shown in Table 3, the content analysis produced 4 themes with their related sub-themes.

#### 3.4.1. Theme of “Benefits for Students”

The participants recognized the efficacy of the educational workshop in acquiring new knowledge and skill in using US in nursing practice:

“*It was a very instructive workshop in that I acquired knowledge of and familiarity with a subject and instrument that is used or could be used during the shift or in the services of a nurse to make some nursing procedures easier*”

(Student 28).

“*This workshop was very useful for understanding the purposes of nursing ultrasound scanning and for understanding how to perform a nursing ultrasound scan*”

(Student 96).

“*Having gained experience with images obtained with an ultrasound scan being able to recognize and identify the separate parts/organs. Before it was all confusing*”

(Student 67).

Besides acquiring new subject matter, the participants identified its efficacy also in integrating theory with practice. One student, for example, stated:

“*I was able for the first time to touch and handle an ultrasound scan. During the internship I was never given the opportunity to use it, often not even the nurses themselves use it as they consider it an instrument solely for physicians. An interesting lesson, an excellent revision of anatomy and good preparation ….*”

(Student 89).

This cultural baggage made the students perceive greater preparation and self-confidence before entering in the clinical learning environments, stimulating their clinical reasoning and curiosity:

“*It was a very instructive workshop in that I acquired knowledge of and familiarity with a subject and instrument that is used or could be used during the shift or in the services of a nurse to make some nursing procedures easier. It was important for me because I acquired knowledge that you normally only learn on the ward. Thanks to this workshop I’ll be going to the ward with a procedure that I can already do*”

(Student 28).

“*Having greater self-confidence in performing some procedures that require greater commitment and difficulty in as short a time as possible ….*”

(Student 2).

“*Taking part in the workshop was very important because it sparked fresh curiosity that I hope over the years to be able to satisfy*”

(Student 64).

“*It allowed me to have an additional path in elaborating clinical reasoning when carrying out procedures; it tends to give you self-confidence and is an instrument that is added to practice for which you are not always a specialist*”

(Student 76).

The perception of the usefulness of the experience is highlighted by the suggestion of many students to implement the training also in future years:

“*Definitely very useful and to be included in the course of study as a workshop even divided over different years, if possible, also for other subjects. It permits a better understanding of anatomy too, and also on the ward for understanding the medical examinations performed*”

(Student 42).

“*Introduction of a new nursing practice, which is a valid help for many procedures, already in basic training to provide an awareness of its use and to encourage it without improvising. A useful experience to be permanently included in the course*”

(Student 98).

The feedback was then of good overall satisfaction, as is clear from this comment:

“*An entirely satisfactory experience*”

(Student 34).

#### 3.4.2. Theme of “Benefits for Patients”

The participants identified benefits that using this instrument can produce in patient care:

“*Possibility of applying the procedure to improve care and ensure a certain comfort for the patient*”

(Student 4).

“*The nursing ultrasound scanning workshop provides appropriate training so as not to go blindly over the patient. The added value is having tried using the instrument on real people who are different to each other*”

(Student 61).

“*Greater knowledge and skill are acquired in using an ultrasound scan that helps ensure more meticulous and complete care for certain categories of patients*”

(Student 13).

#### 3.4.3. Theme of “Benefits for the Profession”

The students believed that acquiring new expertise concerning nursing ultrasound scanning can increase the prestige, growth and autonomy of the nursing profession:

“*Having learned new and more technical knowledge about our care setting. To give greater prestige to our work. A very instructive and fun experience*”

(Student 86).

“*Developing additional skills for the role of nurse allows increasingly greater growth and autonomy for the nursing profession*”

(Student 11).

#### 3.4.4. Theme of “Benefits for the Professional Future”

At the same time, the benefits of skills acquired were also highlighted for the professional future, in terms of gratification, opportunity for interprofessional cooperation, and expendability in the world of work:

“*This workshop added value to a theme that nowadays is increasingly more important and innovative. It is an excellent resource that definitely has positive sides, and I think that after graduation getting to know how to handle it is gratifying and helpful*”

(Student 95).

“*Having added a skill to my personal baggage, thus being able as nursing staff to locate the main sites for performing a diagnostic examination. In addition, knowing how to distinguish a vein from an artery, the bladder and the vena cava helps, in my opinion, to have a better collaboration with the physician too*”

(Student 75).

“*It is an essential workshop to be added to our degree course also for being more independent as recent graduates*”

(Student 45).

### 3.5. Student’s Satisfaction

114 out of 118 participants (97%) assured that they were satisfied with the training they received. Specifically, 102 participants said they were very satisfied (86.5 percent), 12 satisfied (10.2 percent), 3 fairly satisfied (2.5 percent), 1 not very satisfied (0.8 percent), and no enrolled students said they were very dissatisfied.

## 4. Discussion

Ultrasound is a diagnostic imaging methodology that, thanks to its rapidity of use, physical and biological non-invasiveness, the application of non-ionizing radiation and the possibility of implementation in different healthcare settings, has achieved considerable success over the last years [1].

The use of US in nursing care has highlighted numerous advantages. In finding venous access, it improves patient safety, the quality of care provided, and reduces both the patient’s suffering and the need for CVADs [39,42], also decreasing the morbidity, mortality and healthcare costs associated with them [11,12,13,19]. Costs are further reduced by the decrease in time taken to find PIV access and the increase in the success rate which also involves less waste of material [39].

The European Society of Anaesthesiology states that the use of US in the identification of arterial vessels reduces both major and minor complications especially in the most critical patients [14].

The use of US also reduces the days of catheterization or the use of the bladder catheter when not necessary, thus reducing the onset of urinary infections and improving patient outcomes [24].

The use of this technology for lung assessment allows nurses trained to improve outcomes in patients with heart failure, allowing them to promptly detect lung congestion to ensure its correct management [27]. Furthermore, US reduces medical visit time, ensuring a consequent improvement in workflow and clinical efficacy [28]. Patient monitoring also improves the assessment of CVI by trained nurses [30], especially to reduce the risks associated with dialysis procedures or to properly manage fluid infusion in critically ill patients [43]. 

Finally, the increase in satisfaction for healthcare professionals that the use of this technology generates should not be forgotten [29].

Given the optimal cost–benefit ratio associated with these practices, the inclusion in nursing curricula of educational programs that promote familiarity with the use of US, starting from basic training, could be a winning strategy to increase the dissemination of this knowledge and skills in the nursing population.

Even short-term courses could be an initial starting point to disseminate this knowledge as much as possible [44,45].

As evidenced by our results, the training on the use of US appears to have been effective in increasing participants’ knowledge and perceived self-efficacy. The data are also supported by the opinions collected from the students, as well as their self-reported satisfaction.

Theoretical knowledge of the use of the ultrasound scan doubled from the pre-test to the post-test, following USTep. Similar results, although with less of a difference between the pre-test and the post-test, were obtained both by the study by Bortman et al. (2019) [46], that highlighted a growth in the knowledge of Certified Registered Nurse Anesthetists, and by the study of Kaganovskaya et al. (2021) [42], that demonstrated how the knowledge of nurse practitioner students increased following a course comprising a theoretical introduction and a practical simulation.

Very encouraging results were also highlighted in relation to the self-confidence of the students concerning the single procedures presented during the workshop. An improvement in self-confidence was highlighted by 2.2 score difference in the post-test compared to the pre-test. The data are in line with those presented in the studies by Edwards and Jones (2018), Amick et al. (2021) and by Yamada et al. (2023) [4,20,47].

The results on satisfaction expressed by 97% of the sample highlight how much students believe implementing knowledge of US is fundamental for their education. In the study by Edwards and Jones (2018), 71.4% of the emergency nurses believed it fundamental to continue offering the educational program on the ultrasound-guided peripheral intravenous catheter [4].

In the open-ended responses, many students highlighted the multiple benefits of the new educational program: the USTep allowed them to acquire knowledge, practical skills and self-confidence on the US, facilitating the development of clinical reasoning and thorough preparation before the clinical internship, in line with the literature [48,49]. Our students proposed to continue this training by extending it to all three years of the course, increasing the time available for exercises with the device to acquire greater manual skills with this instrument.

Many students highlighted the benefits for patients, in terms of comfort and improvement in the quality of care, since US both improve the efficacy of the technique of inserting a PIV [3,15,16,17,18,19] and prevent urinary tract infections [24,25,26].

This expansion of the skills acquired according to the students can foster the prestige, growth and autonomy of the nursing profession, which could boost the attractiveness of the nursing profession that remains exceedingly low [50,51]. The participants believed that the acquisition of these skills would foster greater cooperation with physicians, professional gratification and greater job opportunities.

### Implications, Limitation, and Future Research

The conducted QI project presents some limitations: monocentric design, lack of a control group and convenience sampling. In addition, self-reported data could introduce potential biases, such as social desirability or overestimation of skills.

Since data collection was performed immediately after the training, little is known about the long-term effectiveness of this educational intervention, particularly regarding knowledge retention and confidence over time.

In contrast, to our knowledge, this is the first study to investigate training on using US for locating multiple areas of the body in a sample of undergraduate nursing students. Currently, published studies involve registered nurses or nurses attending post-basic courses of education. Future research could evaluate not only self-reported data but also the skills of the students. Furthermore, an expansion of the training offered in this area, along the three-year study path, increasing not only the hours of practical exercises, but also the contents (i.e., introduction of the retrieval of PIV accesses with a biological simulator), would allow us to understand how knowledge, self-confidence and skills are maintained in the long term. Finally, further studies could evaluate the actual frequency of application of these methods by students in clinical learning environments, as well as their performance in real-world clinical settings, and ultimately, the impact on patients.

## 5. Conclusions

This quality improvement project shows an ultrasound technology educational program that combines theoretical introduction with simulation training in a three-hour educational workshop, improved nursing students’ knowledge, self-confidence and satisfaction.

These outcomes were confirmed and enriched by the qualitative data; for the students, the training they received was an opportunity for personal growth, could improve the care provided to patients, lessen their suffering and shorten waiting times, without forgetting the potential appreciation of the nursing profession. Considering the very encouraging results, this may serve as the basis for promoting innovation in nursing education, and nursing programs should consider the importance and feasibility of this training and include it in the formal curriculum.

## Figures and Tables

**Table 1:** Pre-test and post-test knowledge scores on 118 nursing students.

Knowledge	Pre-Test N (%)	Post-Test N (%)	Pearson’s χ2 Test *p*-Value
**What is the Graphical Representation of Ultrasounds?**			
▪ Pie chart	11 (9.3%)	1 (0.85%)	χ2 = 201.793 *p* < 0.00001
▪ Bullet graph	6 (5.1%)	0 (0.85%)	
▪ Sinusoidal graph *	84 (71.2%)	116 (98.3%)	
▪ Histogram	17 (14.4%)	0 (0%)	
**What is the Frequency of the Ultrasounds Used in Medical Diagnostics?**			
▪ 0.03 kHz	10 (8.5%)	4 (3.4%)	χ2 = 149.072 *p* < 0.00001
▪ 1.5 kHz	92 (78%)	12 (10.2%)	
▪ 800 kHz	12 (10.2%)	7 (5.9%)	
▪ 1500 kHz *	4 (3.3%)	95 (80.5%)	
**What Aspects Make Nursing Ultrasound Scanning an Effective Method?**			
▪ Physically and biologically non-invasive with no ionizing radiation	0 (0%)	0 (0%)	χ2 = 0.338 *p* = 0.95
▪ Possibility of using multiple care settings	2 (1.7%)	0 (0%)	
▪ Repeatability	1 (0.85%)	1 (0.85%)	
▪ All the above *	115 (97.5%)	117 (99.15%)	
**What Type of Probe do We Use to Get to Find a Vascular Access?**			
▪ Convex	47 (39.84%)	3 (2.5%)	χ2 = 92.666 *p* < 0.00001
▪ Linear *	43 (36.44%)	113 (95.8%)	
▪ Sector	13 (11.01%)	1 (0.85%)	
▪ Microconvex	15 (12.71%)	1 (0.85%)	
**Which of These is Not a Feature of Pelvic Nursing Ultrasound Scanning?**			
▪ It lets us determine the volume of the bladder and its content	11 (9.3%)	3 (2.5%)	χ2 = 94.997 *p* < 0.00001
▪ It simplifies choosing the most suitable bladder catheter	49 (41.5%)	4 (3.4%)	
▪ It makes inserting a bladder catheter easier	20 (16.9%)	1 (0.85%)	
▪ It has diagnostic purposes *	38 (32.2%)	110 (93.2%)	

* The items highlighted in bold correspond to the correct answers.

**Table 2:** Pre-test and post-test self-confidence scores on 118 nursing students.

Self-Confidence	Pre-Test M (SD)	Post-Test M (SD)	Paired *t*-Test *p*-Value
I am confident that my procedure for locating the vein with the aid of the ultrasound scan is correct	1.71 (1.03)	3.87 (0.83)	−23.582 <0.001
I am confident that my procedure for locating the bladder with the aid of the ultrasound scan is correct	1.79 (1.05)	4.06 (0.89)	−20.508 0.008
I am confident I am correctly identifying the probe for assessing veins	1.73 (1.07)	4.08 (0.89)	−22.925 <0.001
I am confident that my procedure for locating the artery with the aid of the duplex ultrasound scan is correct	1.92 (1.06)	4.08 (0.89)	−21.620 <0.001
I am confident that my procedure for locating the inferior vena cava with the aid of the ultrasound scan is correct	1.35 (0.77)	3.42 (1.03)	−21.050 <0.001

**Table 3:** Themes and sub-themes of nursing students’ experiences with US.

Themes	Sub-Themes
Benefits for students	Knowledge Ability Self-confidence Integration of theory-practice Pre-training preparation Clinical reasoning Curiosity
Benefits for patients	Patient’s comfort Better care
Benefits for the profession	Prestige Growth Autonomy
Benefits for the professional future	Professional gratification Interprofessional collaboration Expendability in the world of work

## Data Availability

The raw data supporting the conclusions of this article will be made available by the authors on request.

## Public Involvement Statement:

No public involvement in any aspect of this QI project.

## Guidelines and Standards Statement:

This is an educational innovation project to improve the quality of training for nursing students.
